# Physiological and Molecular Analysis of Aluminium-Induced Organic Acid Anion Secretion from Grain Amaranth (*Amaranthus hypochondriacus* L.) Roots

**DOI:** 10.3390/ijms17050608

**Published:** 2016-04-30

**Authors:** Wei Fan, Jia-Meng Xu, He-Qiang Lou, Chuan Xiao, Wei-Wei Chen, Jian-Li Yang

**Affiliations:** 1 College of Resources and Environment, Yunnan Agricultural University, Kunming 650201, China; fanwei1128@aliyun.com; 2 State Key Laboratory of Plant Physiology and Biochemistry, College of Life Sciences, Zhejiang University, Hangzhou 310058, China; xujiamengican@163.com (J.-M.X.); louheqiang@126.com (H.-Q.L.); 3 Institute of Resource Biology and Biotechnology, Department of Biotechnology, College of Life Science and Technology, Huazhong University of Science and Technology, Wuhan 430074, China; xiaochuanapple@163.com; 4 College of Life and Environmental Sciences, Hangzhou Normal University, Hangzhou 310036, China; 15858223807@163.com

**Keywords:** aluminium, citrate, grain amaranth, oxalate, suppression subtractive hybridization

## Abstract

Grain amaranth (*Amaranthus hypochondriacus* L.) is abundant in oxalate and can secrete oxalate under aluminium (Al) stress. However, the features of Al-induced secretion of organic acid anions (OA) and potential genes responsible for OA secretion are poorly understood. Here, Al-induced OA secretion in grain amaranth roots was characterized by ion charomatography and enzymology methods, and suppression subtractive hybridization (SSH) together with quantitative real-time PCR (qRT-PCR) was used to identify up-regulated genes that are potentially involved in OA secretion. The results showed that grain amaranth roots secrete both oxalate and citrate in response to Al stress. The secretion pattern, however, differs between oxalate and citrate. Neither lanthanum chloride (La) nor cadmium chloride (Cd) induced OA secretion. A total of 84 genes were identified as up-regulated by Al, in which six genes were considered as being potentially involved in OA secretion. The expression pattern of a gene belonging to multidrug and toxic compound extrusion (MATE) family, *AhMATE1*, was in close agreement with that of citrate secretion. The expression of a gene encoding tonoplast dicarboxylate transporter and four genes encoding ATP-binding cassette transporters was differentially regulated by Al stress, but the expression pattern was not correlated well with that of oxalate secretion. Our results not only reveal the secretion pattern of oxalate and citrate from grain amaranth roots under Al stress, but also provide some genetic information that will be useful for further characterization of genes involved in Al toxicity and tolerance mechanisms.

## 1. Introduction

Aluminium (Al) toxicity is one of the major constraints for crop production in acid soils, which occupies approximately 50% of potentially arable lands worldwide [1]. The primary visible symptom of Al toxicity is the inhibition of root elongation, which occurs even at micromolar concentrations of Al [2]. A number of possible mechanisms responsible for the Al-induced inhibition of root elongation have been proposed. For example, Al may alter cytoskeletal structure, interfere with DNA replication, disrupt signal transduction pathways, and trigger oxidative stress response [3,4,5]. Transcriptional analysis also revealed that hundreds to thousands of genes were up- and down-regulated within several hours of exposure to Al stress, which are involved in a variety of physiological and molecular processes [6,7,8,9,10]. Thus, the inhibition of root elongation appears to be a toxic syndrome that results from disorder of a series of physiological and biochemical processes.

On the other hand, different plant species or cultivars within the same species have evolved mechanisms to deal with Al toxicity in the acidic soils. Al-induced organic acid anions (OA) secretion has been well established as a very important Al resistance mechanism in a variety of plant species [4]. Among the various secreted OA in response to Al stress, extensively reported are three major species, *i.e.*, malate, citrate, and oxalate. Over the past decade, genes involved in Al-activated malate secretion and Al-activated citrate secretion have been isolated in a number of plant species [11]. However, genes involved in Al-activated oxalate secretion have not been reported in any plant species.

Secretion of oxalate from plant roots in response to Al stress has been reported in several plant species such as taro (*Colocasia esculenta*) [12], common buckwheat (*Fagopyrum esculentum*) [13], spinach (*Spinacia oleracea*) [14], *Polygonum* species [15], and tea plants (*Camellia sinensis*) [16]. Being a C4 dicotyledonous plant, Grain amaranth (*Amaranthus hypochondriacus* L.) belongs to the *Amaranhaceae* family [17]. Because of the high protein content of the leaves and seeds, and the high levels of the essential amino acid, lysine in the protein, grain amaranth has been becoming one of the world’s most promising foods among health-conscious consumers [17]. In addition, grain amaranths are known to be tolerant to adverse environmental conditions, including drought, acidic, poor and/or saline soils and pests, which can be attributed to their high levels of stored carbon reserves in stem and roots, high water use efficiency, and accumulated compatible solutes and hormones, such as jasmonic acid (JA) [18,19,20,21]. Our previous study showed grain amaranth contains a substantial amount of oxalate and secretes oxalate from roots while suffering from Al stress [22]. Although circumstantial evidence implied that oxalate secretion under Al stress from some oxalate accumulators might be a common feature [22], the physiological and molecular characterization of Al-induced organic acids secretion from grain amaranth roots has not been conducted.

In screening of Al-responsive genes in plants, a number of molecular approaches have been adopted such as differential display reverse transcription-polymerase chain reaction [23], cDNA-amplified fragment length polymorphisms [24], microarray [25], suppression subtractive hybridization (SSH) [26], and mRNA sequencing [9,10,27]. SSH method is a powerful technique to identify multiple differentially expressed sequence tags (EST) in different mRNA populations, and has been used to identify Al-responsive genes in wheat (*Triticum aestivum*) [28], common bean (*Phaseolus vulgaris*) [29], alfalfa (*Medicago sativa*) [30], and rice bean (*Vigna umbellata*) [31].

In the present study, we analyzed both types and patterns of OA secretion from grain amaranth roots in response to Al stress. The results showed that grain amaranth roots secrete both oxalate and citrate into rhizosphere under Al stress, but secretion pattern differed between oxalate and citrate. Using SSH method, we identified a total of 84 ESTs that were up-regulated by 9 h of Al stress. Finally, 6 genes participating in ion transport process were evaluated with respect to their potential role in Al-induced OA secretion.

## 2. Results

### 2.1. Characterization of Aluminium (Al)-Induced Organic Acid Anions (OA) Secretion from Grain Amaranth Roots

**Figure 1:**
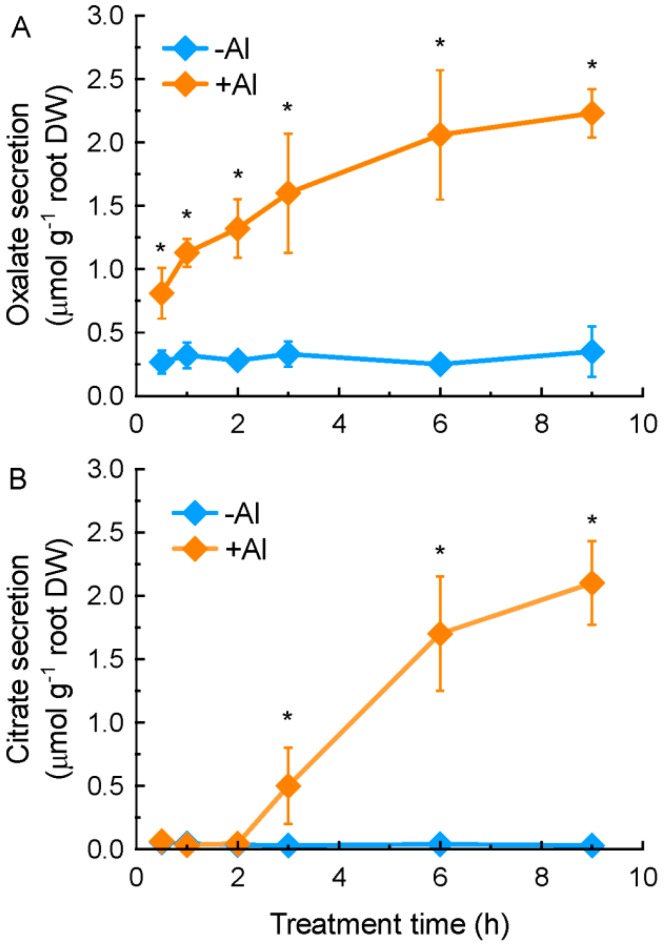
Time-course of aluminium (Al)-induced oxalic acid (**A**) and citric acid (**B**) secretion from grain amaranth roots. Three-week-old seedlings were exposed to 0.5 mM CaCl_2_ solution (pH 4.5) containing 0 or 25 µM Al. Root exudates were collected at indicated intervals for determination of organic acids. Data are means ± SD (*n* = 3). Asterisks indicate that mean values are significantly different between Al treatment and control (Student’s *t* test, *p* < 0.05).

We have previously demonstrated that some oxalate accumulators can secrete oxalate from their roots in response to Al stress [22]. In this present study, we characterized secreted OA profiles and patterns in an oxalate accumulator, grain amaranth (*Amaranthus hypochondriacus* L.). Al treatment rapidly (within 30 min of exposure) stimulated the secretion of oxalate from grain amaranth roots (Figure 1A), although oxalate was detected in the root exudates of grain amaranth without Al treatment (Figure S1). During the entire treatment period, the secretion rate of oxalate was at similar levels (Figure 1A). On the other hand, citrate was not detected in the root exudates until 3 h of exposure to 25 µM Al. The amount of secreted citrate was significantly increased after 6 h of exposure. In the absence of Al stress, citrate was almost undetectable (Figure 1B).

**Figure 2:**
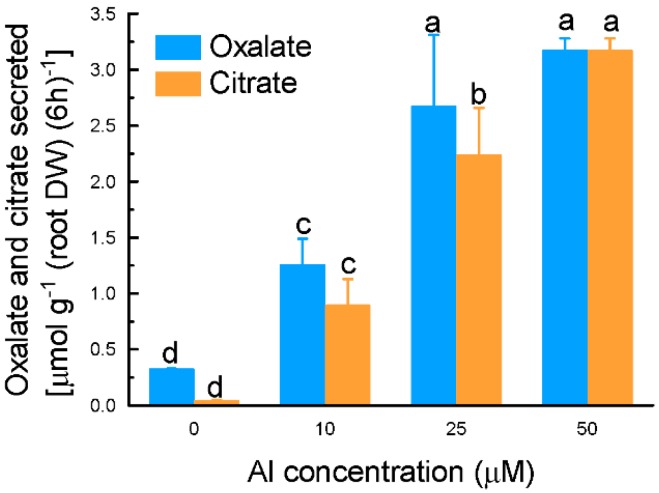
Effect of different Al concentrations on oxalic acid and citric acid secretion from grain amaranth roots. Three-week-old seedlings were exposed to 0.5 mM CaCl_2_ solution containing 0, 10, 25, or 50 µM Al for 6 h. Root exudates were collected for determination of organic acids. Data are means ± SD (*n* = 3). Different letters indicate statistically significant differences (Tukey test, *p* < 0.05).

The secretion of both oxalate and citrate was in a likely dose-dependent manner. However, there is no significant difference in secreted oxalate between 25 and 50 µM Al treatments (Figure 2).

**Figure 3:**
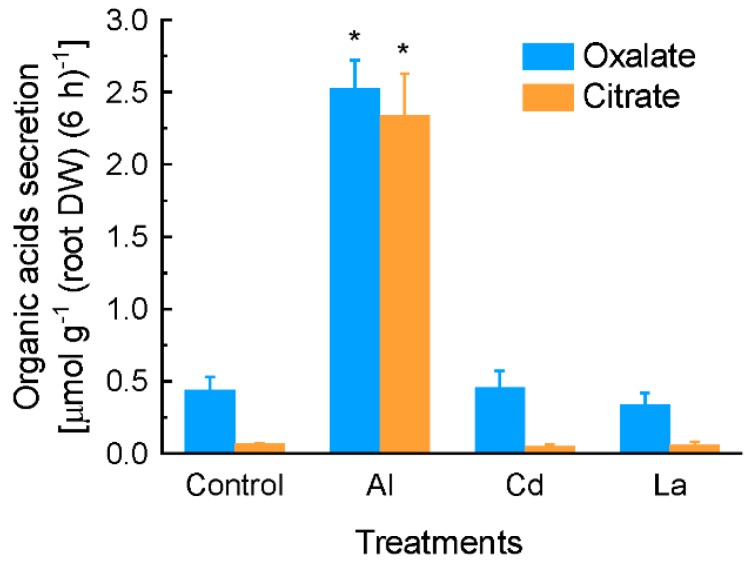
Effect of lanthanum chloride (La) and cadmium chloride (Cd) on the secretion of organic acids from grain amaranth roots. Three-week-old seedlings were exposed to 0.5 mM CaCl_2_ solution containing 25 µM Al, 25 µM La, 10 µM Cd, or without metals as control. Root exudates were collected by 6 h for determination of organic acids. Data are means ± SD (*n* = 3). Asterisks indicate that mean values are significantly different between treatment and control (Student’s *t* test, *p* < 0.05).

When grain amaranth roots were exposed to lanthanum chloride (La) or cadmium chloride (Cd) stress for 6 h, both oxalate and citrate secretion were not induced. In contrast, exposure of roots to 10 µM Al induced secretion of both oxalate and citrate significantly (Figure 3).

### 2.2. Identification of up-Regulated Genes by Al Stress in Amaranth Roots

In order to identify genes potentially associated with Al-induced OA secretion, a forward cDNA library (9 h Al treatment *vs.* control) was constructed. A total of 84 differentially expressed transcripts were obtained, and were grouped into several functional categories according to the Gene Ontology (GO) biological process defined by the TAIR and UniProt database (Figure S2). Among these functional categories, the one that encompasses genes involved in “metabolism and energy” is most predominant. For example, genes encoding malate dehydrogenase, succinate dehydrogenase, and citrate synthase were observed (Table S1). In addition, up-regulation of eight genes involved in hormone metabolism and signaling transduction pathways confirming the recent observation that hormone may partly share Al signal to regulate Al tolerance mechanism such as OA secretion [32,33]. However, this present study was restricted to the genes which could be potentially involved in the transport processes of OA. Therefore, a total of nine genes involved in ion transport process were selected for further. Among these candidates, genes encoding ADP, ATP carrier protein, plasma membrane intrinsic protein (PIP2;1), and inorganic phosphate transporter were excluded, because they have somewhat defined functions that are not associated with OA secretion (Table S1). Finally, six genes including one gene encoding a multidrug and toxic compound extrusion (*AhMATE1*) protein, one gene encoding tonoplast dicarboxylate transporter (*AhTDT*), and four genes encoding ATP-binding cassette (ABC) transporters (*AhABCG11*, *AhABCG21*, *AhABCA2*, and *AhABCB21*, respectively) were chosen for further expression analysis by qRT-PCR (Table 1).

### 2.3. Expression Patterns of Selected Genes Encoding Transporter Proteins

**Figure 4:**
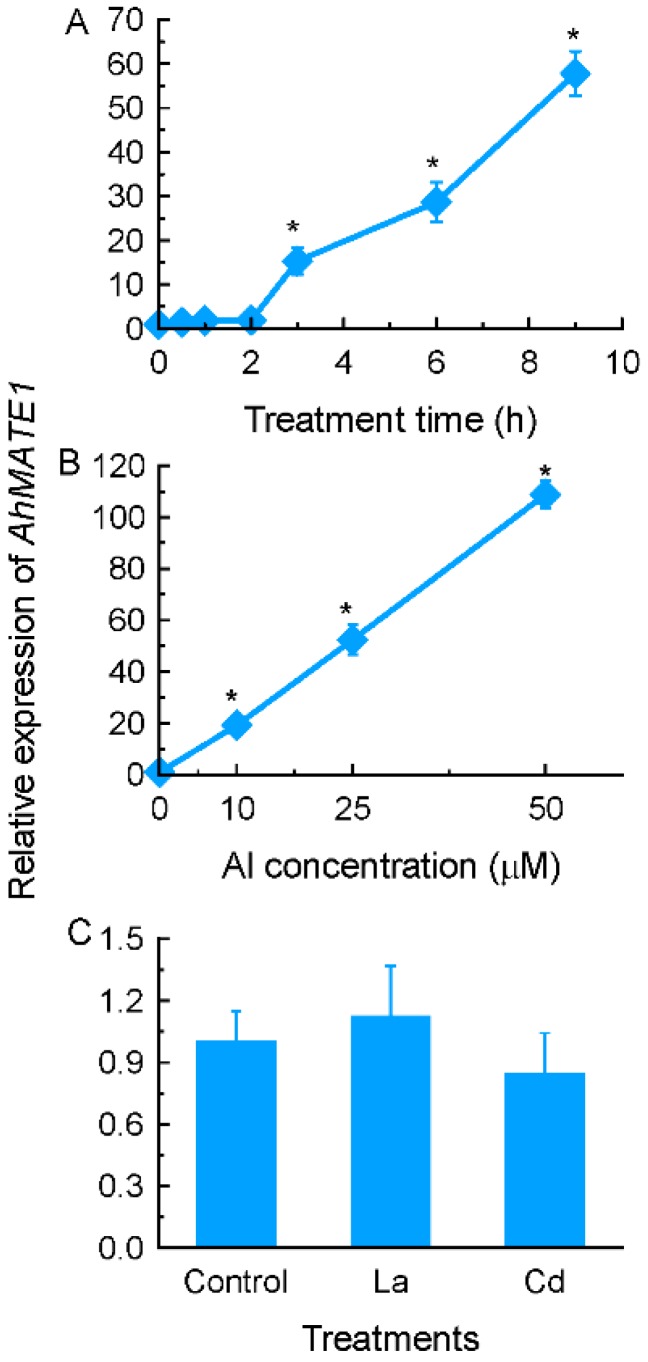
Grain amaranth roots *AhMATE1* gene expression analysis. (**A**) Time-course analysis of *AhMATE1* expression treated with 25 µM aluminium (Al) after various periods by real-time PCR; (**B**) dose response analysis of *AhMATE1* expression treated with different Al concentrations for 9 h; (**C**) expression of *AhMATE1* in response to 25 μM La or 10 μM Cd for 9 h. Total RNA was extracted from roots and qRT-PCR was performed for each gene with glyceraldehyde-3-phosphate dehydrogenase (*GAPDH*) as an internal control. Data are means ± SD (*n* = 3). Asterisks indicate that mean values are significantly different between treatment and control (Student’s *t* test, *p* < 0.05).

A subgroup of MATE proteins has been reported to be associated with Al-activated citrate transporter in some plant species [11]. Thus, it is likely that the gene encoding MATE protein could be involved in Al-induced citrate secretion from grain amaranth roots. In order to verify whether *AhMATE1* is possibly involved in Al-induced citrate secretion, we characterized the expression pattern of *AhMATE1*. The expression of *AhMATE1* is in a time- and dose-dependent manner in response to Al stress (Figure 4A,B), which is in line with citrate secretion pattern (Figure 1 and Figure 2). However, its expression was not affected by La or Cd stress (Figure 4C).

**Figure 5:**
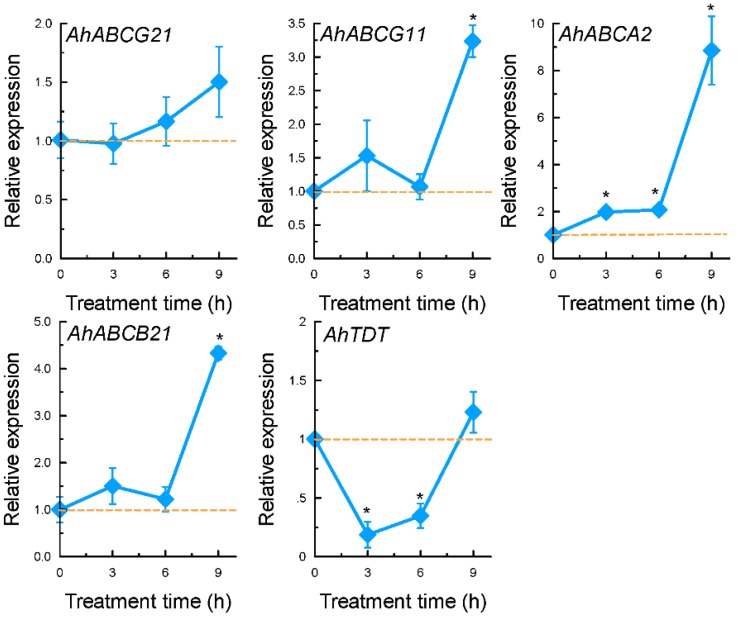
Temporal expression pattern of *AhABCG21*, *AhABCG11*, *AhABCA2*, *AhABCB21*, and *AhTDT* in response to 25 µM Al. Three-week-old seedlings were exposed to 0.5 mM CaCl_2_ solution (pH 4.5) containing 0 or 25 µM Al. Total RNA was extracted from roots and qRT-PCR was performed for each gene with *GAPDH* as an internal control. Data are means ± SD (*n* = 3). Asterisks indicate that mean values are significantly different between treatment and control (Student’s *t* test, *p* < 0.05). Dash line indicates expression level of 1.

**Figure 6:**
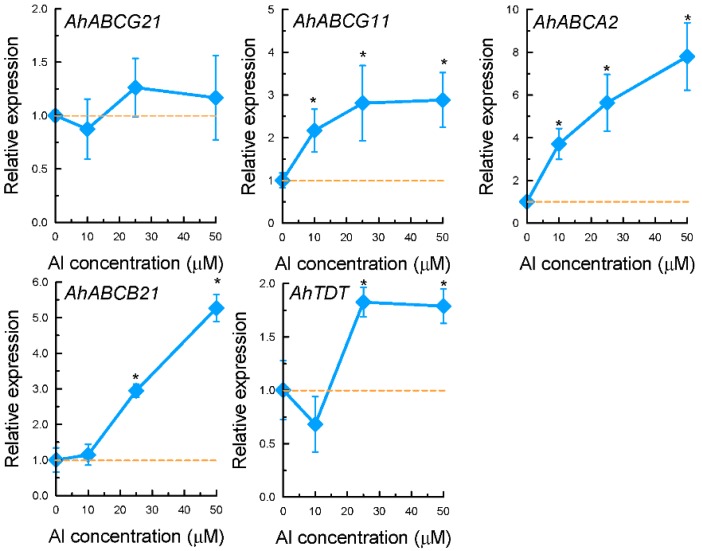
Dosage expression pattern of *AhABCG21*, *AhABCG11*, *AhABCA2*, *AhABCB21*, and *AhTDT* in response to 0, 10, 25, or 50 µM Al for 9 h. Three-week-old seedlings were exposed to 0.5 mM CaCl_2_ solution (pH 4.5) containing different concentrations of Al or not. Total RNA was extracted from roots and qRT-PCR was performed for each gene with *GAPDH* as an internal control. Data are means ± SD (*n* = 3). Asterisks indicate that mean values are significantly different between treatment and control (Student’s *t* test, *p* < 0.05). Dash line indicates expression level of 1.

**Figure 7:**
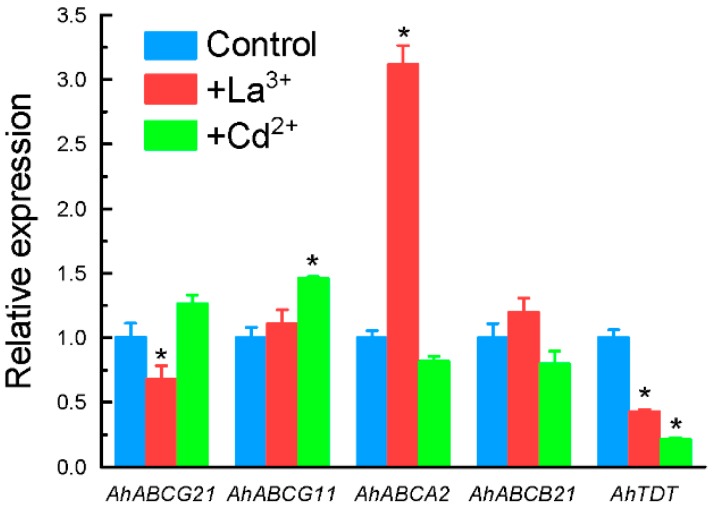
Effect of La and Cd treatment on expression of *AhABCG21*, *AhABCG11*, *AhABCA2*, *AhABCB21*, and *AhTDT* in grain amaranth roots. Three-week-old seedlings were exposed to 0.5 mM CaCl_2_ solution containing 25 µM La, 10 µM Cd for 9 h, or without metals as control. Total RNA was extracted from roots and qRT-PCR was performed for each gene with *GAPDH* as an internal control. Data are means ± SD (*n* = 3). Asterisks indicate that mean values are significantly different between treatment and control (Student’s *t* test, *p* < 0.05).

Among the four genes encoding ATP-binding cassette (ABC) transporters, the expression of *AhABCG21* was not significantly affected by Al stress in both time-course and dose-response experiments (Figure 5 and Figure 6). The expression of *AhABCG11* and *AhABCB21* were only significantly increased after 9 h of exposure, whereas that of *AhABCA2* was increased within 3 h of exposure (Figure 5). The expression of both *AhABCA2* and *AhABCB21* displayed dose-dependent increase in response to Al stress (Figure 6). The expression of *AhABCG11*, however, reached the maximum at 10 µM Al, and cannot be further increased with increased Al concentrations (Figure 6). The expression of these four genes was not affected by either La or Cd stress with the exception of *AhABCA2*, which was greatly induced by La treatment, and *AhABCG21*, which was inhibited by La (Figure 7).

The expression of a gene encoding tonoplast dicarboxylate transporter, *AhTDT*, was observed to be down-regulated within 6 h of exposure to 25 µM Al, and slightly increased after 9 h of exposure (Figure 5). Compared to control conditions, exposure to Al at 10 µM for 9 h slightly inhibited the expression of *AhTDT*, and higher Al concentrations resulted in a slight increase in expression levels (Figure 6). Both La and Cd significantly inhibited its expression (Figure 7).

## 3. Discussion

Many plant species secrete OA from roots to neutralize Al toxicity [4,34]. Here we characterized the secretion of OA from grain amaranth roots under Al stress, and some unique features are highlighted. First, roots of grain amaranth secrete both oxalate and citrate in response to Al stress (Figure 1 and Figure 2). While some plant species secrete only a single type of OA into the rhizosphere under Al stress, others secrete two types of OA simultaneously. For example, Al-induced specific secretion of citrate in *Cassia tora* [35], malate in wheat [36], and oxalate in buckwheat [13] was observed. Both citrate and malate were secreted from rye (*Secale cereale*) roots when exposed to Al stress [37]. Evidence for a similar situation has been demonstrated in Arabidopsis [38] and some Al-tolerant wheat genotypes [39]. However, this study is the first report on simultaneous secretion of oxalate and citrate from grain amaranth roots. Second, the secretion pattern differed between oxalate and citrate. Based on the timing of secretion, OA secretion can be grouped into two patterns [34,40]. In pattern I, organic acids were immediately secreted after the beginning of Al stress, whereas in the pattern II, an induction period ranging from several hours to several days are required for the initiation of OA secretion. The rapid secretion and similar secretion rate of oxalate during the entire exposure period indicated that oxalate secretion from grain amaranth roots fits pattern I response (Figure 1A), whereas increased secretion rate of citrate over time indicated that it belongs to pattern II response (Figure 1B). Increased evidence showed that patterns I and II operate independently during activated and regulated process [34], suggesting complexity in secretion of OA from grain amaranth in response to Al. Thirdly, the amount of Al-induced oxalate secretion is far more than citrate secretion at 2 h, but both of them were similar almost while treated Al more than 6 h (Figure 1), suggesting that the key role of oxalate in detoxifying Al toxicity at early stage. Finally, the secretion of both oxalate and citrate exhibited specificity to Al stress since other metals failed to stimulate the secretion (Figure 3). In tomato, La stress failed to induce the secretion of oxalate [41]. Cd stress, however, stimulated the secretion of oxalate in a time- and dose-dependent manner [42]. In both *Cassia tora* and rice bean, Al specifically induced citrate secretion from their roots [35,43]. The specificity of secretion to Al stress indicates that secretion of both OA from grain amaranth roots is regulated by specific transport pathways rather than by a common stress response.

Using the SSH method, we have identified a total of 84 genes that are up-regulated by Al stress in grain amaranth roots (Table S1). These genes were functionally categorized into several physiological and molecular events (Figure S2). For example, Al stress triggered the expression of genes involved in “metabolism and energy” which are most predominant in our library. Similarly, a previous study in rice bean suggested that metabolic changes act as an adaptive mechanism for plants to deal with Al toxicity [31]. The importance of mitochondrial metabolism in Al resistance has recently been summarized [44]. Here, up-regulation of a set of genes involved in tricarboxylic acid cycle points to the possible contribution of citrate metabolism to its secretion (Table S1). However, the key genes encoding enzymes that are involved in the oxalate biosynthesis are still poorly understood. It has been reported that oxalate content is not related to the amount of secretion in amaranth plants, because its content is far more than secreted [22]. Recent genome-wide transcriptome analysis of Al-responsive genes in common buckwheat and tartary buckwheat also found that oxalate metabolism is not a limiting factor of Al-induced oxalate secretion [9,10].

Our data showed that a gene-encoding MATE protein, *AhMATE1*, is a potential citrate transporter in grain amaranth. MATE proteins represent a large family of transporters in prokaryotes and eukaryotes [45]. For example, there are 58 and at least 40 MATE orthologs in *Arabidopsis* and rice, respectively. The role of MATE proteins in Al resistance has been reported in a number of plant species such as sorghum (*Sorghum bicolor*) [46], barley (*Hordeum vulgare*) [47], wheat [39], rice (*Oryza sativa*) [48], *Arabidopsis* [38], rice bean [49], and *Eucalyptus camaldulensis* [50]. It has been concluded that citrate-transporting MATE proteins are characteristic of having 12 transmembrane domains and one highly conserved amino acid sequence in the cytosolic loop between the second and third transmembrane domains [49]. Amplification of *AhMATE1* full length by RACE PCR revealed that it shares all conserved structures with other known citrate transporters (Data not shown). Furthermore, the expression pattern of *AhMATE1* fit the secretion pattern of citrate. Therefore, it is very likely that this gene is responsible for Al-induced citrate secretion from grain amaranth roots.

Among 9 cDNA clones identified as ion transporters, nearly half of them belong to the family of ABC transporters (Table S1). ABC transporter represents a very large gene family. Members of this family can be found in all taxa, and shuttle a broad range of substrates such as lipids, phytohormones, carboxylates, heavy metals, chlorophyll catabolites and xenobiotic conjugates [51]. In fact, experimental evidence has pointed to the role of ABC transporters in transporting OA in plants. For example, *AtABCB14* has been reported to be implicated in transporting of malate from apoplast into guard cells [52]. Knockout of *AtPDR6*, a member of the pleiotropic drug resistance protein (PDR) subfamily of ABC transporters, resulted in significant decrease of OA (3-hydroxypropionic acid, succinic acid, fumaric acid, and malic acid) in *Arabidopsis* root exudates [53]. These results raised the question as to whether ABC transporter is involved in Al-dependent oxalate secretion. In the present study, although both *AhABCB21* and *AhABCA2* exhibited at least partly time- and dose-dependent expression patterns in response to Al stress (Figure 5 and Figure 6), they did not correlate well with the pattern of Al-dependent oxalate secretion. In addition, the expression of *AhABCA2* was also significantly induced by La stress. In wheat, the induction of *TaMDR1*, a member of ABC transporters, was demonstrated to be associated with Al toxicity, and some calcium channel inhibitors including La could induce the expression of *TaMDR1* [54]. It is possible that up-regulation of *AhABCA2* might be involved in Al toxicity rather than Al resistance.

While whether ABC transporters are involved in Al-induced OA secretion remains unknown, emerging evidence has suggested the importance of this gene family in other plant Al resistance mechanisms. In a screening to identify genes involved in *Arabidopsis* Al resistance, both *AtALS1* and *AtALS3* have been found to contribute to Al detoxification. *AtALS1* is a tonoplast-localized half-type ABC transporter, and predicted to function to transport chelated Al into vacuoles [55]. However, *AtALS3* encodes a transmembrane domain of a bacterial ABC transporter, and may function to redistribute accumulated Al away from sensitive tissues [56]. Huang *et al.* (2009) identified two rice genes, *OsSTAR1* and *OsSTAR2*, with the former encoding the nucleotide-binding domain and the later encoding the transmembrane domain of one ABC transporter [57]. Interestingly, OsSTAR1 and OsSTAR2 directly interact with each other to form a functional transporter complex, which possibly transports UDP-glucose to mask cell wall composition to protect the binding of Al [57]. In the present study, expression of some ABC transporter genes in response to Al stress was regulated, suggesting that these genes should have roles in adaptation of grain amaranth to Al toxicity. However, the detailed function of each member of the ABC transporters identified in this study has to be characterized in the future study.

In the present study, the expression of a gene encoding tonoplast dicarboxylate transporter was examined. Oxalate is the simplest dicarboxylate. Thus, transporters involved in oxalate secretion are permeable to dicarboxylates. Ryan and Delhaize (2010) hypothesized that transporters responsible for Al-induced malate or citrate secretion might be evolved from other malate or citrate transporters that originally performed different functions [58]. It is therefore likely that mutations might occur in the coding region of the tonoplast dicarboxylate transporter which alters its subcellular localization and functions. Although this EST was enriched in our forward library, the expression of this gene was only slightly increased at higher Al concentrations with relatively long exposure time (Figure 6). The inconsistency between Al-induced oxalate secretion pattern and the expression pattern of *AhTDT* precludes it being a potential candidate for oxalate transporter. However, it remains possible that Al mediates oxalate secretion through post-translational regulation of the oxalate transporter gene. For example, in both wheat and barley, *TaALMT1* and *HvAACT1* are constitutively highly expressed in Al-resistant genotypes, and Al directly or indirectly activates their functions [28,47].

## 4. Materials and Methods

### 4.1. Plant Materials and Growth Conditions

Grain amaranth (*Amaranthus hypochondriacus* L.) seeds were soaked in de-ionized water overnight. Then the seeds were placed on the filter paper moistened with 0.5 mM CaCl_2_ solution at pH 4.5 and kept in the dark at 26 °C. Germinated seeds were transferred to a net tray, which was floated on a 5 L of 0.5 mM CaCl_2_ solution at pH 4.5 in a plastic container. The seedlings were kept in the dark at 26 °C for 7 days, and then transferred to a controlled-environment growth room with a 14 h/26 °C day at a light intensity of 300 μmol·photons·m^−2^·s^−1^ and 10 h/22 °C night regime. The solution was renewed daily. At day 4, the seedlings were transplanted into 1.2 L plastic pots (four holes per pot, 2 seedlings for each hole) containing aerated nutrient solution. One-fifth-strength Hoagland nutrient solution was used consisting of the following macronutrients in mM: KNO_3_, 1.0; Ca(NO_3_)_2_, 1.0; MgSO_4_, 0.4; NH_4_H_2_PO_4_, 0.2, and the following micronutrients in μM: NaFeEDTA, 20; H_3_BO_3_, 3; MnCl_2_, 0.5; CuSO_4_, 0.2; ZnSO_4_, 0.4; (NH_4_)_6_Mo_7_O_24_, 1. The solution was adjusted to pH 4.5 with 1 M HCl and renewed every 3 days.

### 4.2. Treatments

Three-week old seedlings were subjected to the following treatments. Prior to each treatment, the roots were rinsed by soaking in aerated 0.5 mM CaCl_2_ solution (pH 4.5) overnight. For the time-course experiment, roots of seedling were exposed to 0.5 mM CaCl_2_ solution (pH 4.5) with or without 25 μM AlCl_3_ for 0.5, 1, 2, 3, 6, or 9 h. For the dose-response experiment, roots of seedling were exposed to 0.5 mM CaCl_2_ solution (pH 4.5) containing 0, 10, 25, or 50 μM AlCl_3_ for 6 or 9 h. For LaCl_3_ or CdCl_2_ treatment, seedlings were exposed to 0.5 mM CaCl_2_ solution (pH 4.5) containing 25 μM LaCl_3_ or 10 μM CdCl_2_ for 6 or 9 h. After treatments, root exudates were collected and immediately used to analyze organic acids, and root tissues were frozen immediately in liquid N_2_ and stored in −80 °C refrigerator until use.

### 4.3. Collection of Root Exudates

Collected root exudates were passed through a cation-exchange resin column (16 mm × 14 cm) filled with 5 g of Amberlite IR-102 B resin (H^+^ form), followed by an anion-exchange resin column (16 mm × 14 cm) filled with 2 g of Dowex 1 × 8 resin (100–200 mesh, formate form). Organic acids retained on the anion-exchange resin were eluted with 15 mL of 1 M HCl, and the eluate was concentrated to dryness by a rotary evaporator (40 °C). The residue was dissolved in 2 mL of Milli-Q water and subjected to organic acid determination.

### 4.4. Measurement of Oxalate and Citrate

Oxalic acid was determined by ion chromatography using an IonPac AS11 anion-exchange analytical column (4 mm × 250 mm) equipped with a guard column (4 mm × 50 mm). The mobile phase was 30 mM NaOH at a flow rate of 0.6 mL·min^−1^. A standard curve was made by analyzing different concentrations of oxalic acid with the same procedure. Citric acid was determined enzymatically [59]. In briefly, to 1 mL of sample solution, 120 µL 1 M Tris/HCl buffer (pH 7.8) and 15 µL 10 mM NADH were added. After incubation at 25 °C for 40 min, 2 µL enzyme mixture (1.25 U lactate dehydrogenase and 0.5 U malate dehydrogenase) was added and the reaction mixture was incubated for an additional 40 min. the change in A340 was monitored after the reaction was initiated with addition of citrate lyase (0.5 U).

### 4.5. RNA Isolation and Construction of SSH Library

Total RNA was extracted using the RNAeasy mini kit (Tiangen, Shanghai, China). cDNA was amplified with Super SMART™ PCR cDNA synthesis Kit (Clontech, Palo Alto, CA, USA). For identification of up-regulated genes, forward subtracted cDNA library was constructed. For this, amplified cDNA from 25 μM Al-treated for 9 h roots was used as the “tester”, while amplified cDNA from control as the “driver”. PCR-select cDNA subtraction was performed according to instruction of PCR-Select™ cDNA Subtraction Kit (Clontech, Palo Alto, CA, USA). The PCR products were sub-cloned into the pGEM-Teasy vector (Promega, Madison, WI, USA) and then transformed into chemically competent *E. coli* (DH5α) cells. Transformed clones were grown on LB plates containing X-gal and ampicillin for blue/white screening. Positive clones were selected and further checked by PCR for the presence of gene inserts after plasmid isolation, and clones with >200 bp single fragment insert were selected for DNA sequencing.

### 4.6. Sequence Homology and Functional Annotation

The cDNA sequences were compared with the GenBank database using the online Basic Local Alignment Search Tool (BLAST) program [60]. The detailed search procedure was the same as our previous report [31].

### 4.7. Quantitative Real-Time PCR

Total RNA was isolated from root tissues as described above. First-strand cDNA was synthesized from 1 µg of total RNA using Superscript™ reverse transcriptase (Takara, Dalian, China). 1 μL (100 ng·μL^−1^) of cDNA in 10 μL volume system were used for quantitative analysis of gene expression performed with SYBR Premix ExTaq (Takara, Dalian, China). The primer pairs for each gene were listed in Table 1, and the PCR amplification conditions were as follows: 94 °C for 5 min; 45 cycles of 94 °C for 10 s, 55 °C for 15 s and 72 °C for 20 s. The endogenous gene glyceraldehyde-3-phosphate dehydrogenase (*GAPDH*) was used as the internal control with the following primers: 5′-TTCGGTCACAGGAACCCAGA-3′ and 5′-ACCTTCTTGGCACCACCCTT-3′. For each target gene, the PCR reactions were carried out in three biological and technical repeats. The relative expression level was calculated by formula 2^–∆∆*C*p^.

### 4.8. Statistical Analysis

Each result shown in the figures was the mean of three replicated treatments, and at least two independent experiments were conducted for each result. The significant differences between treatments were statistically evaluated by Students’s *t* or Tukey test method.

## 5. Conclusions

We characterized Al-induced OA secretion from grain amaranth roots and observed simultaneous secretion of oxalate and citrate in response to Al stress with the secretion pattern differed. A total of 84 genes up-regulated by Al stress were identified in grain amaranth roots, and several genes encoding transporter proteins were highlighted. Although identification of oxalate transporter remains a great challenge, the present results provide some genetic information that will be useful for further characterization of genes involved in plant Al tolerance and toxicity mechanisms.

## Figures and Tables

**Table 1:** List of selected cDNA clones associated with ion transport processes and primer pairs used for qRT-PCR analysis.

Gene Name ^a^	Annotation	Primer Pairs	Amplicon Size (bp)
*AhABCG21*	ABC transporter G family member 21	for: AGGTGACTTGCCTATGGAACT	107
		rev: TCGTAAGGGTAAGGATAAATG	
*AhABCG11*	ABC transporter G family member 11	for: AAACACACTTTCTTCAATCCCAT	205
		rev: ACCCGTTATGATACCCATTAGAA	
*AhABCA2*	ABC transporter A family member 2	for: ACATCGCAAGACAAGCCG	116
		rev: CCCCACATACCTGGCTCC	
*AhABCB21*	ABC transporter B family member 21	for: TGCTATGGGGGAGAAGGT	122
		rev: AAAGGGGTATGGACGAAA	
*AhTDT*	Tonoplast dicarboxylate transporter	for: TACAGCGACTTCCGACGACTA	267
		rev: ACAAGCAACAAAGAACACCCC	
*AhMATE1*	MATE protein	for: GGTCCTTTGGTGCTCCTGC	163
		rev: CCACTGACACCCAAACGACAT	

^a^ Gene name was temporarily assigned to the selected cDNA clones according to their closest homologous genes.

## Supplementary Materials

Supplementary materials can be found at http://www.mdpi.com/1422-0067/17/4/608/s1.
