# Supplementary material for: Physiological and Molecular Analysis of Aluminium-Induced Organic Acid Anion Secretion from Grain Amaranth (Amaranthus hypochondriacus L.) Roots
Source: Int J Mol Sci. 2016 Apr 30;17(5):608. doi: 10.3390/ijms17050608 (PMC4881440; doi:10.3390/ijms17050608)
Supplement: Supplementary file 1 [file ijms-17-00608-s001.zip › ijms-121910-Supplementary Figures-publish.docx]

**Supplementary Materials: Physiological and Molecular Analysis of Aluminium-Induced Organic Acid Anion Secretion from Grain Amaranth
(*Amaranthus hypochondriacus* L.) Roots**

Wei Fan, Jia-Meng Xu, He-Qiang Lou, Chuan Xiao, Wei-Wei Chen and Jian-Li Yang





**Figure S1.** Ion charomatograghy profiles of oxalic acid detection in root exudates. (**A**) Root exudates collected from 0.5 mM CaCl_2_ solution for 3 h; (**B**) root exudates collected from 0.5 mM CaCl_2_ solution containing 25 μM Al for 3 h.


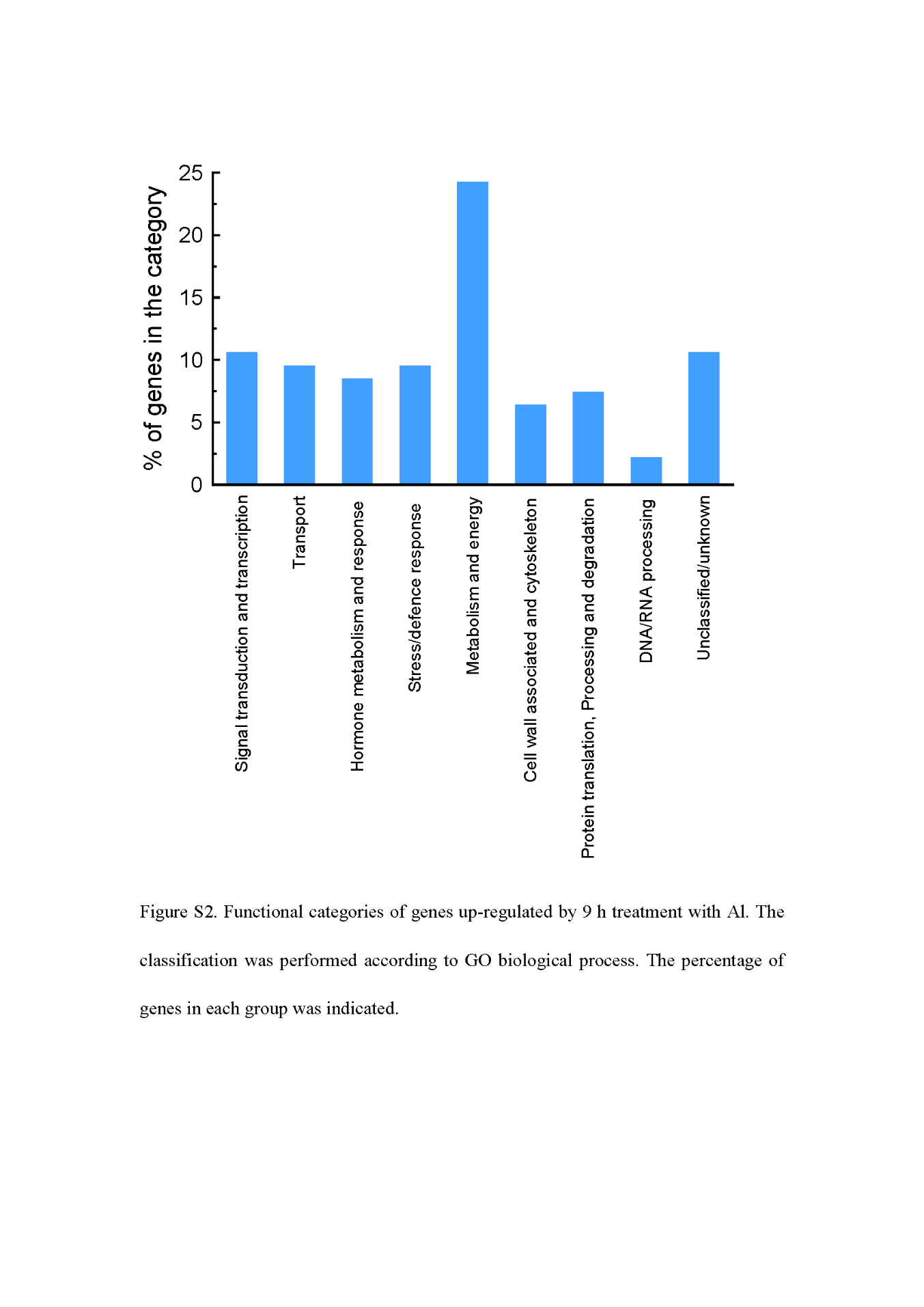


**Figure S2.** Functional categories of genes up-regulated by 9 h treatment with Al. The classification was performed according to Gene Ontology (GO) biological process. The percentage of gene in each group was indicated.
